# Female mating rates and their fitness consequences in the common house spider *Parasteatoda tepidariorum*

**DOI:** 10.1002/ece3.9678

**Published:** 2022-12-28

**Authors:** Apostolos Angelakakis, Natascha Turetzek, Cristina Tuni

**Affiliations:** ^1^ Behavioral Ecology, Faculty of Biology Ludwig‐Maximilians‐University Munich Planegg‐Martinsried Germany; ^2^ Evolutionary Ecology, Faculty of Biology Ludwig‐Maximilians‐University Munich Planegg‐Martinsried Germany

**Keywords:** fitness, mating behavior, monandry, polyandry, Theridiidae

## Abstract

Mating systems, with varying female mating rates occurring with the same partner (monandry) or with multiple mates (polyandry), can have far reaching consequences for population viability and the rate of gene flow. Here, we investigate the mating rates of the common house spider *Parasteatoda tepidariorum* (Theridiidae), an emerging model for genetic studies, with yet undescribed reproductive behavior. It is hypothesized that spiders belonging to this family have low re‐mating rates. We paired females twice with the same male (monandry) or with different males (polyandry), and recorded behaviors, mating success and fitness resulting from single‐ and double‐matings, either monandrous or polyandrous. Despite the study being explorative in nature, we predict successful matings to be more frequent during first encounters, to reduce female risk of remaining unmated. For re‐mating to be adaptive, we expect higher fitness of double‐mated females, and polyandrous females to experience highest mating success and fitness if reproductive gains are achieved by mating with multiple partners. We show that the majority of the females did not mate, and those that did mated only once, not necessarily on their first encounter. The likelihood of re‐mating did not differ between monandrous and polyandrous encounters and female mating experience (mated once, twice monandrous, twice polyandrous) did not affect fitness, indicated by similar offspring production. Female twanging of the web leads to successful matings suggesting female behavioral receptivity. Cannibalism rates were low and mostly occurred pre‐copulatory. We discuss how the species ecology, with potentially high mating costs for males and limited female receptivity, may shape a mating system with low mating rates.

## Graphical abstract

The study uncovers the reproductive behavior of the emerging spider model *Parasteatoda tepidariorum*. Mating rates are low, with most females mating once, and double‐matings did not yield fitness benefits. Female vibratory signaling is key for mating, and events of female sexual cannibalism occur before mating. The high mating costs for males may shape a mating system characterized by low mating rates.

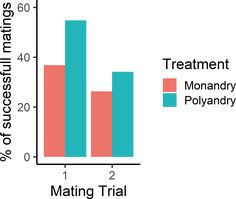

## 1 INTRODUCTION

Mating systems are primarily shaped by the frequency of mating in each of the sexes, determined by life history and ecological (i.e., socio‐demographic) factors (Andersson, 1994; Emlen & Oring, 1977). Female mating rates, whether occurring repeatedly with the same partner (monandry) or with multiple mates over a single reproductive cycle (polyandry), can have far reaching consequences for population viability and the rate of gene flow (Holman & Kokko, 2013; Lumley et al., 2015; Pizzari & Wedell, 2013). Polyandry appears to be especially common and widespread (Taylor et al., 2014), and is reported also in socially monogamous species (Westneat & Stewart, 2003). This has challenged the traditional concept of choosy and monogamous females (Bateman, 1948), diverting theoretical and empirical efforts to explain the evolution and maintenance of polyandry (Arnqvist & Nilsson, 2000; Jennions & Petrie, 2000). The main adaptive explanations focus on females receiving direct fitness benefits through increased access to resources (e.g., food, protection; Arnqvist & Nilsson, 2000) and viable sperm (Reinhardt & Ribou, 2013; Sutter et al., 2019), and/or indirect benefits to their offspring by avoiding genetic incompatibility and male genotypes of inferior quality (Jennions & Petrie, 2000; Simmons, 2005), or both (Fedorka & Mousseau, 2002; Tuni et al., 2013). Females may also derive direct benefits by accepting multiple matings from reducing the costs associated with male harassment (i.e., convenience polyandry; Boulton et al., 2018). The magnitude of these benefits needs to outweigh the costs associated to mating (e.g., disease transmission, injury, time and energy; Knell & Webberley, 2004; McNamara et al., 2008) for females to solicit re‐matings with novel males. Sexual conflict may, on the other hand, represent a non‐adaptive route to polyandry where females are forced into a suboptimal number of matings by males (Arnqvist & Rowe, 2013; Parker, 2006).

Despite being ubiquitous, polyandry is highly variable, with extreme numbers of mating partners being reported between and within species (Gowaty, 2013; Taylor et al., 2014). For example, honeybee queens *Apis mellifera* mate on average with 12 males during one mating flight (Tarpy et al., 2004) and the wasp spider *Argiope bruennichi* with maximum of two (Weiss et al., 2020); in a Spanish population of the field cricket *Gryllus bimaculatus* all females were found to be polyandrous (Bretman & Tregenza, 2005), whereas for bumble bees (*Bombus* spp.) 20% of colonies were found to be sired by more than one father (Payne et al., 2003). Ecological constraints, such as limited encounter rates between the sexes due to female‐biased sex ratios, high mate‐search costs for males and/or low population densities, may overall select for low levels of polyandry (Berger‐Tal & Lubin, 2011; Emlen & Oring, 1977; Tuni & Berger‐tal, 2012). Interestingly, in the absence of paternal care, as in most arthropods, low mating rates are considered to be seldom female‐driven (Kokko & Mappes, 2013). However, female reluctance in re‐mating may instead reflect male manipulation of female behavior (Hosken et al., 2009). For example, females of many species enter a refractory period after their first mating that is driven by seminal fluid molecules transferred by males at mating (Avila et al., 2011; Chapman & Davies, 2004). The duration of such a period can be substantial (e.g., 30 days for the mosquito *Aedes aegypti*; Degner & Harrington, 2016), and in short lived animals this may potentially affect lifetime reproduction, leading to single fathered offspring. Male‐controlled mating rates can be further supported by documenting the fitness benefits from experimentally inducing polyandry in monogamous females (Arnqvist & Andres, 2006; Baer & Schmid‐Hempel, 1999). If, on the other hand, individuals do not have the opportunity to mate multiply and/or the costs associated with polyandry are high, females may maximize their fitness by mating only once, and selection should favor monogamy (Klug, 2018).

Most studies testing for adaptive explanations of polyandry estimate fitness benefits of re‐mating with same or novel males (Evans & Magurran, 2000; Simmons, 2005; Slatyer et al., 2012; Tregenza & Nina, 1998). To fully understand how mating systems evolve and are maintained, studies should also investigate whether re‐mating rates are under male or female control by including the study of behaviors at mating for both sexes, together with the fitness consequences of mating decisions. Disentangling whether mate acceptance (or rejections) depends on female choosiness or male manipulation is challenging. Yet studying the outcome of sequential encounters may help shed light on these dynamics. Individuals may behave differently at each encounter, and male–female interactions and the fitness outcomes may largely depend on the outcome of previous encounters (Whittingham & Dunn, 2010). Altogether, these factors may ultimately affect the total number of mating partners in a female's lifetime.

Spiders represent excellent model organisms to investigate mating system evolution, being well‐studied in the field of sexual selection (Eberhard, 2004; Elias et al., 2011; Huber, 2005; Tuni et al., 2020). Most of our knowledge on spider mating rates derives from experimental studies (reviewed in Tuni et al., 2020), which show that spider mating systems are highly variable, ranging from monogamous (e.g., certain species of wolf spiders; Jiao et al., 2011; Norton & Uetz, 2005) to highly polyandrous species (e.g., the Palearctic nuptial feeding spider; Toft & Albo, 2015). High mating rates may often remain undetected in laboratory studies as females may become sexually reluctant to re‐mate after their first mating (Aisenberg et al., 2009; Elgar & Bathgate, 1996; Uetz & Norton, 2007), even for extended periods of time (Perampaladas et al., 2008). Such decrease in receptivity could indicate increased choosiness in already‐mated females or male manipulation of the females' physiology (Aisenberg & Costa, 2005). The large variation in spiders' mating systems, alongside being one of the most specious taxonomic groups on earth—with 50.114 reported species (World Spider Catalog, 2022)—calls for more studies comprehensively analyzing species mating behaviors and their fitness outcomes.

In this study, we investigate the mating system of the common house spider *Parasteatoda tepidarorium* (previously, *Achaearanea tepidariorum*) of the Theridiidae family (Theridiinae subfamily; Liu et al., 2016). Benefiting from its small size, relatively short generation time and the ability to be raised in large populations under laboratory conditions, *P. tepidariorum* is currently one of the best studied spiders in genetic and developmental biology studies (Hilbrant et al., 2012; Oda & Akiyama‐Oda, 2020). The molecular experimental methods (e.g., expressional analysis, gene knockdown, overexpression and comparative transgenesis) as well as genomic and transcriptomic data available are unparalleled by any other spider (Hilbrant et al., 2012; Janeschik et al., 2022; Oda & Akiyama‐Oda, 2020; Posnien et al., 2014; Schwager et al., 2017; Turetzek et al., 2016). Yet, surprisingly little is known on the species reproductive behavior, although accessible genomic resources and functional gene validation methods would make it an excellent model to understand the genetic basis of spider mating behavior. Male–female interactions at mating have been rarely studied so far (Ma et al., 2022), with records being rather anectodical (i.e., based on observations of only three pairs; Knoflach, 2004). Males are active and reach sedentary females on their webs, generally staying on the fringe of it, putatively guarding the female from other males or waiting to approach and mate (Ma et al., 2022). In this species, there is a marked sexual dimorphism, with male body mass being 10 times smaller than females' (Oda & Akiyama‐Oda, 2020). Males mate (i.e., hereafter the term mating and copulation are synonyms used to indicate transfer of sperm) with only few and fast insertions of the reproductive organ (i.e., pedipalps) only lasting seconds (Knoflach, 2004). Records on *P. tepidarorium* document damage of male pedipalps, but mated males are able to nevertheless father offspring, suggesting that the functionality of the pedipalp is not compromised (Locket & Luczak, 1974). This often leads to characterizing the species as absent of genital mutilation (Miller, 2007). While there is lack of visible mating plugs (Knoflach, 2004; Ma et al., 2022) the presence of a small apical portion of the male pedipalp was reported inside the female reproductive tract, with its functional role yet unknown (Abalos & Baez, 1963). Additionally, the rate of sexual cannibalism from laboratory raised females appear to be relatively low (11%–13%) for the species (Ma et al., 2022).

It is hypothesized that females belonging to this spider family have low re‐mating rates, because males are generally short lived and suffer high mate search costs for sedentary females (Segoli et al., 2006), and the timeframe of female receptivity is relatively short (Knoflach, 2004). We therefore tested whether these general predictions also apply to our study species. We paired *P. tepidarorium* females twice with the same male (monandry) or with different males (polyandry), and scored (1) mating behaviors, to describe courtship and mating interactions, (2) mating success, to estimate mating rates, and (3) fitness consequences of single‐ and double‐matings, the latter being either monandrous or polyandrous, to shed light on the adaptive nature of the species mating system. Although our study is exploratory in nature, we predict successful matings to be more frequent during first encounters with males, to reduce the risk of remaining unmated, and less frequent during subsequent encounters. For re‐mating to be adaptive, we expect higher fitness of double‐mated females. Specifically, if females gain indirect benefits by mating multiply with different partners, polyandrous matings should be most frequent and yield the highest fitness outcome (i.e., higher hatching success of the brood). If females gain direct benefits (i.e., sperm supply) by mating more than once, we expect no difference in mating success and fitness outcome (i.e., number of offspring) of polyandrous and monogamous matings, but fitness of double‐mated females to be higher than for single mated females. We also tested for the effect of individual body mass (here used as a proxy for body size) in predicting successful mating outcomes, as it is a trait that generally plays an important role in female mating decisions, as shown for other spider species (Aisenberg, 2009; Maklakov et al., 2004) and specifically Theridiids, where larger males, known to perform more intense vibratory courtship (Sivalinghem & Mason, 2021), are also those preferred by females (Stoltz et al., 2008). We also investigated the effect of female body mass in affecting fitness, as being generally correlated with fecundity in arthropods (Roff, 2002).

## 2 MATERIALS AND METHODS

### 2.1 Collecting and rearing spiders

Spiders were either captured from several buildings located in Munich (Germany) during March and April 2021 or taken from laboratory stock derived from the original genome line (Schwager et al., 2017; received from Göttingen; Janeschik et al., 2022). The laboratory stock is a genetically homogenous isofemale strain that was inbred for at least 40 generations, and originally collected in Göttingen, Lower Saxony, Germany. This allowed reaching an adequate sample size of 183 spiders, 125 originating from the genome line (hereafter, G) and 58 from wild‐caught females (hereafter, W), and variation in origin was accounted statistically. All spiders were raised from egg emergence to adulthood in the laboratory at room temperature (approx. 23°C) under natural light conditions. Cocoons laid by wild caught and/or lab raised females, were collected 2 days after production. These were placed in a new vial covered with a styrofoam plug and equipped with tissue paper on the bottom moistened three times a week. After hatching, spiderlings were fed with approximately 20–25 fruit flies (*Drosophila* spp.). Ten days later, spiderlings were separated into individual vials (2.5 × 9.5 cm) and were fed five fruit flies, three times per week. Once juveniles (second to third instar), 10 fruit flies were provided with the same frequency. Females that reached a subadult stage (i.e., fifth instar) were transferred to bigger vials (5 × 10 cm) and given one to two house flies (*Musca domestica*) three times per week throughout their adulthood, while males were instead given seven to eight fruit flies three times per week throughout their adulthood. Sexes of this species can only be distinguished once they undergo several (five to six) molting stages (Quade et al., 2019), and become sexually dimorphic. Females retain a larger abdomen (i.e., opisthosoma) and remain stationary on webs, while males, which are smaller in size, possess a slimmer, red‐colored body, develop a pair of thick pedipalps and display active walking behavior.

### 2.2 Mating trials

Spiders were randomly assigned to mating trials 1–8 weeks post maturation to adulthood. There was no significant difference in age at testing (*N* days from adulthood to 1st trial; mean) of females (*N* days ± SE, range; monandry 17.35 ± 0.92, 10–38, *n* = 40; polyandry 17.22 ± 1.03, 10–36, *n* = 41; *t*‐test, log‐transformed, *t* = −0.76, df = 1, *n* = 81, *p* = .44), but males assigned to the polyandrous treatment were unintentionally younger than those in the monogamous (monandry 26.26 ± 2.14, 11–56, *n* = 38; polyandry 16.86 ± 1.81, 10–58, *n* = 29; *t*‐test, log‐transformed, *t* = −3.47, df = 1, *n* = 67, *p* = .001*), which we controlled for statistically (see below). Before testing, male vials were inspected for the presence of a sperm web, which generally appeared as a horizontal web sheet on the styrofoam plug of the vial. Male copulatory organs in spiders are not connected to the testes, and through the process of sperm induction, males release sperm on sperm webs and uptake them in their pedipalps by dipping these in the sperm droplet. This process is known to last for relatively long in *P. tepidarorium* (Knoflach, 2004) and is not repeated during copulation. It was observed only occasionally, hence the presence of the web was used as a proxy to consider sperm induction completed in males.

In order to limit stressful manipulation and damage of female webs, we measured individual body mass, to the nearest 0.001 mg, 2 days before the first mating trial using a semi‐micro digital scale (Mettler Toledo). Females were exposed sequentially to two males, once per day with a 1‐day break: they were either given the same male twice (monanadry treatment, *n* = 40 females) or two different males (polyandry treatment, *n* = 44 females). The males matched the female's social experience, meaning two naïve spiders were paired in trial 1, and two experienced spiders (i.e., that had encountered the opposite sex, regardless of the outcome of the interaction) on trial 2, with males from the polyandrous treatment being used with two different females. This was done to standardize previous experience for both sexes. In the case of sexual cannibalism, where the female killed and consumed the male before mating, the latter was replaced with a novel male during the subsequent encounter (trial 2) to complete the double mating trial design. If cannibalism occurred during trial 1, the female was assigned to a polyandrous treatment. If occurring during trial 2, we did not replace the male and considered the trial concluded. Therefore, males were tested one to two times each, with the exception of one male used three times. Individuals assigned to a mating pair were never from the same cocoon and from same mothers to avoid inbreeding (i.e., crossing siblings). Animals derived from G were 60 females and 65 males, and those from W were 24 females and 34 males. Pairs consisted of G females paired twice with G (39.3%, *n* = 33) or W (25%, *N* = 21) males, and W females paired twice with G (22%, *n* = 19) and W (8%, *n* = 7) males, while in four cases these mated with both W and G (6.7%).

Trials were conducted by placing the male inside the female's housing vial, by lifting the lid of the vial on one side and gently pushing the male inside with a paintbrush. The vial was then located inside a custom‐made chamber with carton side walls and top to limit environmental disturbance. A light‐source was placed stationary on the back side to allow video recordings of male and female interactions. A GoPro Hero9‐Camera was placed in front of the vial at a fixed distance of 20 cm. All trials were recorded for a total of 20 min, a timeframe justified by previous reports on courtship duration in this species falling within this time range—range duration of pre‐copulatory courtship 6–25 min, mean 17.3 min, *n* = 3 (Knoflach, 2004), and an average of 155.5 s, *n* = 20 (Ma et al., 2022)—and not least by logistics (i.e., the need to test a high number of animals). Occurrence of cannibalism and mating were noted by the observer (A.A.). After the trial, males were returned to their housing vial.

### 2.3 Fitness

Once females completed the two mating trials, regardless of trials' outcome, females were monitored daily for cocoon production for 4 consecutive weeks. Females of this species lay up to 10–12 cocoons, but within the timeframe of our study they produced a maximum of four cocoons.

When laid, cocoons were collected in standard glass test tubes and the wrapped silk was opened after 5 days using Dumont #5 pair of forceps (Fine Science Tools). Eggs were kept in test tubes with a small piece of wet paper on the lid. These were checked daily if they have reached the postembryonic stage (approximately 180 h after laying of the cocoon at 25°C; Mittmann & Wolff, 2012). After hatching, the total number of spiderlings, unhatched eggs, as well as the “unsuccessful” eggs, − shrunken smaller eggs compared with the others eggs with a light‐yellow color—were counted using a stereomicroscope (Zeiss). This allowed estimating the proportion of hatched eggs per cocoon.

### 2.4 Video scoring of mating behaviors

We scored videos of the mating trials to qualitatively and quantitatively describe male and female mating behaviors using the software BORIS. During male–female interactions, males generally approach the female on her web by walking towards the female, and performing tapping movements with front legs, seemingly in response to certain female leg movements and vibrations on the web. Specifically, the female repeatedly bounces her abdomen and, synchronously or not, lifts legs I and II to pluck web strings, a behavior defined as twanging in other Theridiids (*Parasteatoda wau*; Lubin, 1986), or simply plucking (Knoflach, 2004). Males approach the female and often interact physically by touching the female with their anterior legs. They may also respond with vibrations (i.e., bouncing of abdomen and/or body rocking) themselves. These behaviors are repeated in multiple sequences until successful pairing occurs. Copulation, where sperm is transferred, is fast, after positioning below the female the male inserts his pedipalp into the female epigyne and within seconds releases his sperm by applying hemolymph pressure, indicated by swelling of the pedipalps tip (Knoflach, 2004). After sperm transfer the male directly moves away from the female.

For each video we scored the following behaviors: (1) latency to twanging (i.e., time from the start of the trial until first female twang); (2) total number of female twangs; (3) latency to first male approach (time from the start of the trial to moving towards the female and making physical contact with front legs); (4) total number of male approaches; (5) total number of attempted matings (i.e., male tries to enter the mating position); (6) occurrence of female cannibalism (i.e., pre or post‐copulatory female consumption of the male); (7) mating (i.e., male enters mating position and inserts pedipalp for successful copulation), and whether mating occurs once or twice during the same trial (i.e., males re‐entering and inserting their pedipalps again, after the first successful copulation); and (8) latency to mating (i.e., time from the start of the trial to successful mating). We did not score bouncing due to the difficulty in scoring such behavior accurately from the videos. Among the total 168 videos, for 31 video‐inspection was not possible (e.g., damaged or missing video‐recordings). In six trials, females failed to build a functional web, instead loosely spinning few silk threads on the bottom of the vial. Therefore, behaviors could not be scored as males failed to approach the female without a web and the female failed to signal to the male. The outcome of matings in the latter was always unsuccessful and the data points excluded from further analyses.

### 2.5 Statistical analyses

To test whether mating success (i.e., successful copulation of the pair), male and female behaviors at mating are affected by the treatment applied, we conducted generalized linear mixed effect models (GLMMs) including as fixed effects treatment (monandry and polyandry), trial order (first and second), their interaction, and relative body mass difference between the sexes (female mass–male mass) used to capture the individual state of both sexes in one variable, which could play a role in male–female interactions. We additionally explored the effect of male body mass alone, replicating these models after replacing relative body mass difference with male mass. Male and female ID were included as random effects. Binomial family distribution was used for mating success, Poisson for latencies. The relative body mass difference and the trial order were grand‐mean‐centered. To specifically ask if the likelihood of mating during trial 2 with same (monandry) or different (polyandry) males, were affected by the spiders' previous mating experience (i.e., outcome of trial 1) we ran a linear model testing for the effect of a previous mating (successful or not), treatment (monandry and polyandry), and body mass difference on the likelihood of successful matings during trial 2. To account for variation in spider origin (wild‐caught, W and laboratory genome line, G) and in male age, which due to logistics was not standardized, we expanded the statistical models described above to include these, and the interaction between W and G, as additional factors.

We tested the correlation between number of female twangs and male approaches using correlation coefficient, as these appeared to be related. Finally, we tested for the effect of female twanging on the outcome of the mating (successful copulation or not) by re‐running the GLMM for analyzing mating success described above, including number of female twangs (the latter was grand‐mean‐centered and normalized with the standard‐deviation). We also tested for the effect of number of female twangs on the likelihood of cannibalism by conducting a GLMM including spider ID to account for repeated measures. We compared the likelihood of cannibalism in monogamous and polyandrous, and first and second trials using Chi‐square tests, and differences in relative body size difference in trials with and without cannibalism were tested using *t*‐test. We also tested if the number of attempted matings differ between mated and unmated pairs using *t*‐test.

To test whether mating once or twice, with the same (twice monandry, M) or different male (twice polyandry, P), affected female fitness we conducted linear models to analyze the effect of mating experience (mated once, twice M, twice P) and female body mass on the likelihood of reproducing (i.e., laying at least one viable cocoon), total number of cocoons laid (0–4), the proportion of viable cocoons (i.e., defined as the number of cocoons with viable spiderlings among the total number of cocoons laid) and the total number of offspring (i.e., the sum of all developed spiderlings across multiple cocoons) produced per female. Generalized linear mixed effect models were used to further explore the effects of treatment (mated once, twice M, twice P), female mass and cocoon number (1–4) on the number of eggs laid per cocoon and the proportion of eggs that hatched per cocoon. Female ID was included as a random effect. The model was further expanded to include spider origin (see above).

All statistical analyses were performed using R (version 4.1.1, https://www.r‐project.org/) and the following packages: lme4, MASS, readr, dbplyr, tidyr, ggplots2, tidyverse.

## 3 RESULTS

### 3.1 Mating rates

**FIGURE 1:**
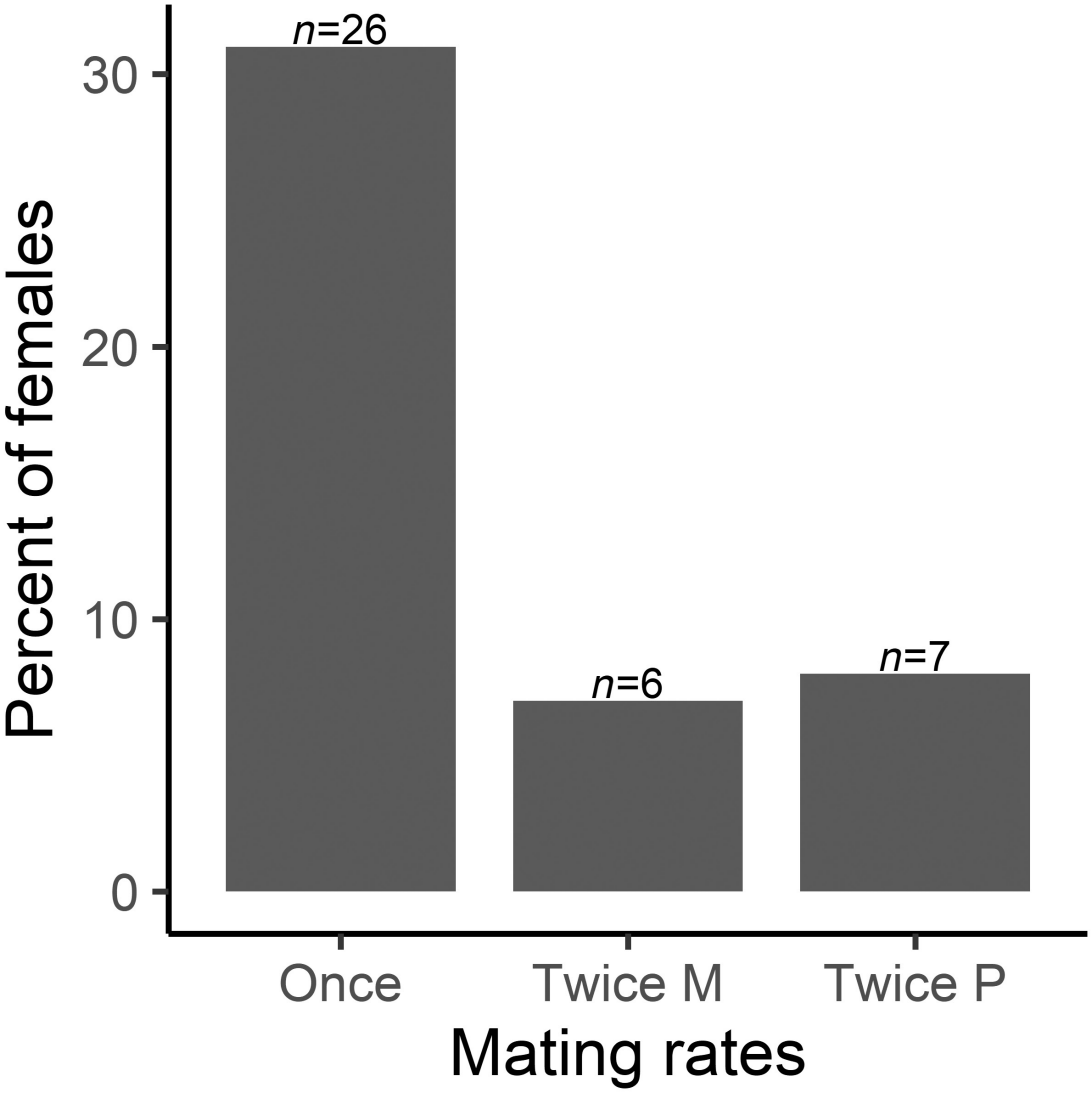
Percentage of females (%) among the 84 tested (*n* = 40, monandry treatment; *n* = 44, polyandry treatment) that successfully mated only once, twice with the same mating partner (twice M = monandry) and twice with a novel mating partner (twice P = polyandry).

**FIGURE 2:**
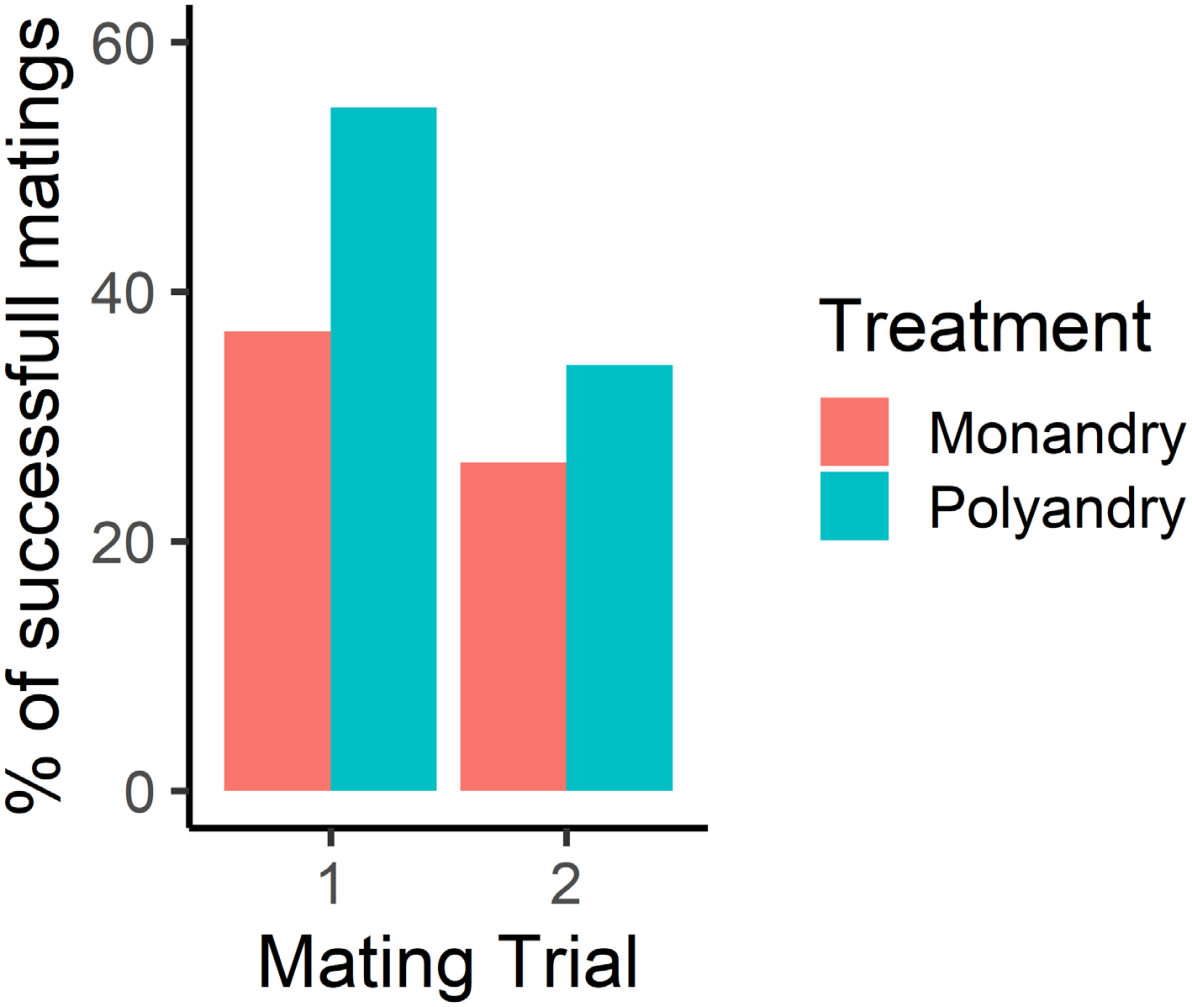
Percentage of successful matings (%) of spiders in monandrous and polyandrous treatments during first encounters between naive individuals (trial 1, number of matings, monandry *n* = 14, polyandry *n* = 23) and subsequent encounters between socially experienced individuals (trial 2, number of matings, monandry = 10, polyandry = 15).

We observed overall low mating rates, as a total of 39 (45.2%) females and 51 (51.5%) males mated successfully (61 trials out of 168). The number of females that mated once was significantly higher than those mated twice (*χ* ^2^ = 4.33, df = 1, *p* < .037; Figure 1). No significant difference in the likelihood of mating successfully was observed between females exposed twice to the same mate (twice monandry, 15%, *n* = 6) or to two novel ones (twice polyandry, 16%, *n* = 7) (*χ* ^2^ = 0.077, df = 1, *p* = .78; Figure 1). The likelihood of mating successfully during a trial was not affected by mating treatment (monandry, 31.5%, *n* = 24; polyandry, 44%, *n* = 38), the order of testing (trial 1, 46.2%, *n* = 37; trial 2, 30.5%, *n* = 25) (Table 1; Figure 2), nor by the relative difference in body mass between the sexes (Table 1).

**TABLE 1:** Results from the GLMMs investigating the effects of mating treatment (polyandry and monandry), trial number (1 and 2), their interaction, and relative body mass difference between the sexes (female–male mass) (model a) and intensity of female twanging behavior (*N* twangs performed) (model b), on the likelihood of mating successfully (GLMM binomial).

	Mating success	
	Model a	Model b
Fixed effects	***β* (95% CI)**	
(Intercept)^a^	−0.87 (−1.42, −0.33)	−1.05 (−1.68, −0.47)
Treatment (polyandry)	0.61 (−0.18, 1.390)	0.47 (−0.44, 1.41)
Mating trial (1, 2)^b^	−0.54 (−1.56, 0.47)	−0.29 (−1.39, 0.73)
Treatment (polyandry) × Mating trial^b^	−0.41 (−1.79, 0.92)	−1.23 (−2.86, 0.41)
Relative body mass^b^	−0.08 (−13.27, 13.77)	−0.70 (−16.95, 15.31)
*N* female twangs^c^		***0.69 (0.23, 1.18)***
Random effects	***σ*** ^**2**^ **(95% CI)**	
Female ID	0.50 (0.37, 0.67)	0 (0, 0)
Male ID	0 (0, 0)	0.32 (0.23, 0.42)

*Note*: Point estimates and 95% credible intervals are shown on a logit scale and relative to the reference category (intercept, monandry, and for the other effects in a standardized level). The residual variance component is fixed to π^2^/3. Significant effects are shown in bold and in italics.

^a^ Reference category, estimate for treatment monandry and mean values of remaining fixed effects.

^b^ Mean centered.

^c^ Mean‐centered and normalized with the standard‐deviation.

The likelihood of mating successfully on trial 2 was not affected by the novelty of the mating partner, whether the same male (monandry) or a novel male (polyandry) or by the outcome of the previous mating trial, whether successful or unsuccessful mating [GLM—binomial, *β* (95% CI); Intercept* −1.23 (−2.09, −0.36); Treatment 0.21 (−0.82, 1.30); Previously mated (yes, no) 0.59 (−0.39, 1.55); Relative body mass −2.74 (−20.42, 15.33)]. Male age and spider origin (W or G), and/or their interaction, did not affect the likelihood of mating nor mating on trial 2 (Table S1), nor did male mass (Table S2).

### 3.2 Behaviors at mating

Pre‐copulatory cannibalism occurred in 17 trials (10.4%) and did not differ between mating treatments (monandry, *n* = 4; polyandry, *n* = 13; *χ* ^2^ = 3.18, df = 1, *p* = .07), testing order (trial 1, *n* = 10; trial 2, *n* = 7; *χ* ^2^ = 0.32, df = 1, *p* = .57). Post‐copulatory cannibalism occurred only once (trial 1, polyandry treatment). The relative difference in body mass between the sexes was higher in trials with non cannibalized males compared with trials in which males were cannibalized (Welch 2 sample *t*‐test, *t* = 2.32, df = 26.48; *p* = .028).

None of the behaviors measured—latency to first female twang, total number of female twangs, latency to first male approach, total number of male approaches, latency to mating (Table 2)—were affected by the mating treatment (monandry and polyandry), nor body mass difference between the sexes (Table 3). In contrast, an interaction between treatment and testing order (trial 1 and 2) affected female behaviors as those of the polyandrous treatment performed twangs later in time and performed less twangs during their second trials. Similarly, males of the polyandrous treatment approached females later in time and less frequently on their 2nd trial (Tables 2 and 3). Male age and spider origin (W or G) did not affect any of the above‐mentioned behaviors (Table S3).

**TABLE 2:** Behaviors measured during male–female interactions, given in mean ± SE, median, range and sample size (*N*) according to the treatment group (monandry and polyandry) and in which mating trial (1 and 2) they were documented.

Behaviors	Monandry		Polyandry	
	Trial 1	Trial 2	Trial 1	Trial 2
Latency to female twanging (s)	137.24 ± 41.02	166.48 ± 46.43	150.48 ± 48.76	280.84 ± 71.58
	50.29	50.9	64.94	73.4
	2.41–1064.53	5.91–1200.62	11.90–1063.08	5.15–1065.73
	32	35	25	25
Total *N* of female twangs	28.5 ± 3.96	25.95 ± 4.29	22.41 ± 4.41	22.07 ± 3.67
	26	13	15	22
	0–106	0–98	0–107	0–61
	36	37	29	27
Latency to 1st male approach	249.35 ± 58.85	279.71 ± 52.86	161.84 ± 55.56	284.21 ± 75.15
	122.77	208.91	81.90	97.91
	7.41–1163.02	8.90–1173.34	4.41–1093.91	19.91–1088.74
	25	28	20	21
Total *N* of male approaches	7.39 ± 1.12	6.08 ± 1.14	7.86 ± 1.60	6.11 ± 1.21
	8	4	7	4
	0–23	0–25	0–34	0–21
	36	36	29	26
Latency to mating (s)	452.15 ± 56.31	524.95 ± 50.39	617.28 ± 53.74	412.41 ± 60.94
	503.17	452.32	581.30	286.18
	33.43–1127.02	202.61–999.98	82.45–1156.74	79.65–997.65
	12	10	12	4

*Note*: Specifically these are: Latency to female twanging (i.e., time from the start of the trial until first female twang); total number of female twangs; latency to first male approach (time from the start of the trial to moving towards the female and making physical contact with front legs); total number of male approaches, as the total number of attempted matings (i.e., male tries to enter the mating position); latency to mating (i.e., time from the start of the trial to successful mating).

**TABLE 3:** Results from the GLMMs investigating the effects of mating treatment (monandry and polyandry), mating trial (1 and 2), their interaction, and relative body mass difference (female–male mass) on male and female behaviors.

	Model estimates *β* (95% CI)				
	Latency to female twanging	Total *N* of female twangs	Latency to 1st male approach	Total *N* of male approaches	Latency to mating
Fixed effects					
(Intercept)^a^	4.34 (3.89, 4.76)	2.90 (2.50, 3.29)	5.03 (4.59, 5.49)	1.45 (1.03, 1.86)	506.56 (342.47, 664.19)
Treatment (polyandry)	0.16 (−0.47, 0.85)	−0.36 (−0.91, 0.22)	−0.19 (−0.89, 0.48)	−0.19 (−0.78, 0.42)	−26.93 (−306.75, 239.10)
Mating trial (1, 2)^b^	0.04 (−0.01, 0.08)	−0.06 (−0.14, 0.04)	***0.08 (0.05, 0.12)***	***−0.25 (−0.43, −0.05)***	61.00 (−237.93, 354.29)
Treatment (polyandry) × Mating trial^b^	***0.78 (0.65, 0.89)***	***−0.41 (−0.63, −0.19)***	***0.87 (0.73, 1.03)***	***−0.45 (−0.84, −0.04)***	−289.19 (−776.26, 194.06)
Relative body mass^b^	−7.03 (−17.67, 4.28)	8.01 (−1.82, 17.97)	0.96 (−10.02, 12.37)	4.16 (−6.15, 14.77)	−3155.99 (−8319.68, 1682.37)
Random effects	***σ*** ^**2**^ **(95% CI)**				
Female ID	0.72 (0.52, 0.96)	0.32 (0.21, 0.44)	0.45 (0.31, 0.64)	0.24 (0.16, 0.33)	0 (0, 0)
Male ID	1.18 (0.92, 1.54)	1.24 (0.95, 1.59)	1.43 (1.07, 1.89)	1.39 (1.06, 1.78)	17,706.46 (8393.57, 31,639.56)

*Note*: Specifically these are: Latency to female twanging (i.e., time from the start of the trial until first female twang); total number of female twangs; latency to first male approach (time from the start of the trial to moving towards the female and making physical contact with front legs); total number of male approaches, as the total number of attempted matings (i.e., male tries to enter the mating position); latency to mating (i.e., time from the start of the trial to successful mating). Point estimates and 95% credible intervals are shown on a logit scale and relative to the reference category (intercept, monandry, and for the other effects in a standardized level). The residual variance component is fixed to π^2^/3. Significant effects are shown in bold and in italics.

^a^ Reference category, estimate for treatment monandry and mean values of remaining fixed effects.

^b^ Mean centered.

**FIGURE 3:**
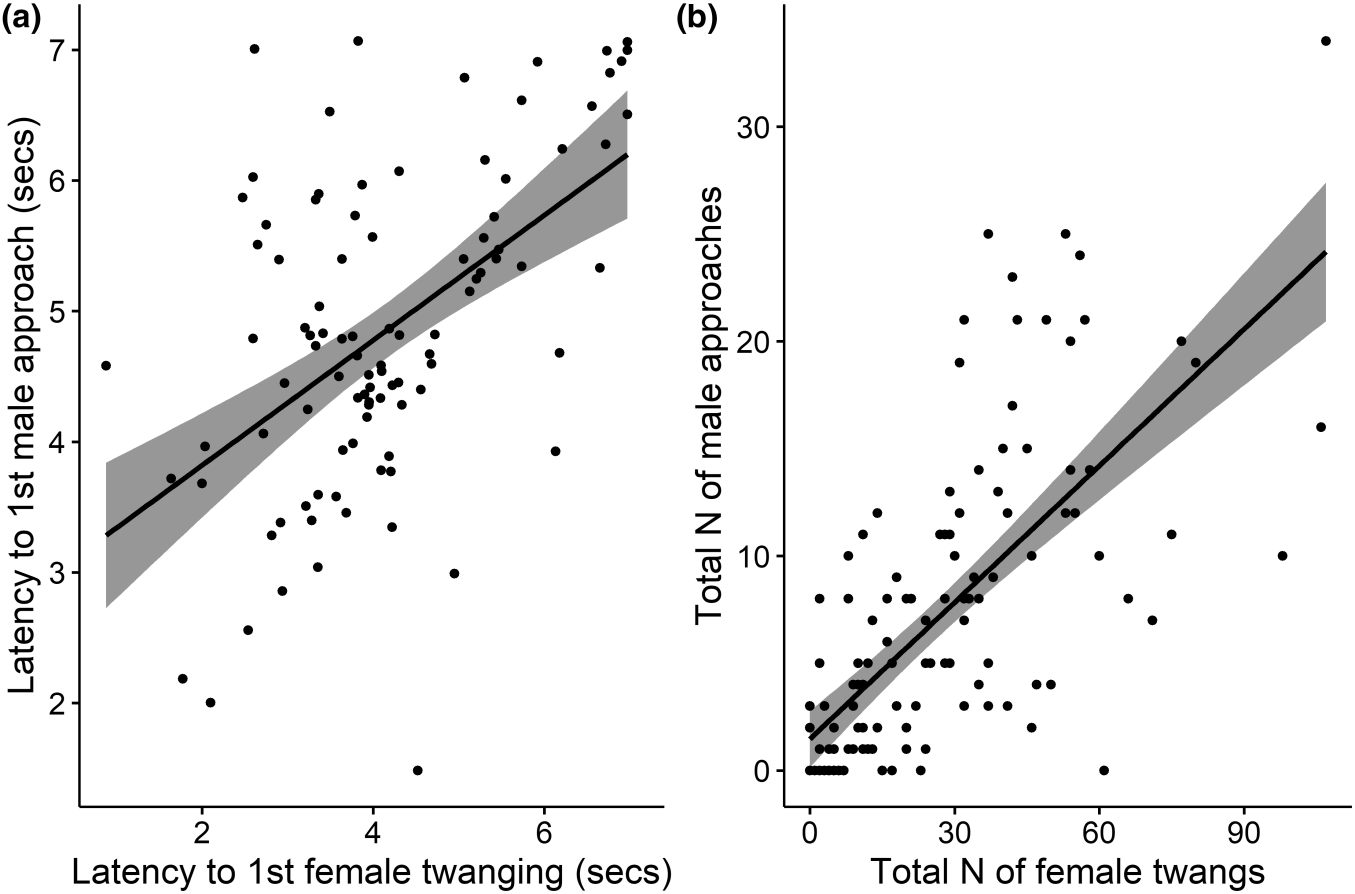
(a) Correlation between the latency to female twanging and latency to first male approach, and (b) correlation between the total number of female twangs and total number of male approaches.

**FIGURE 4:**
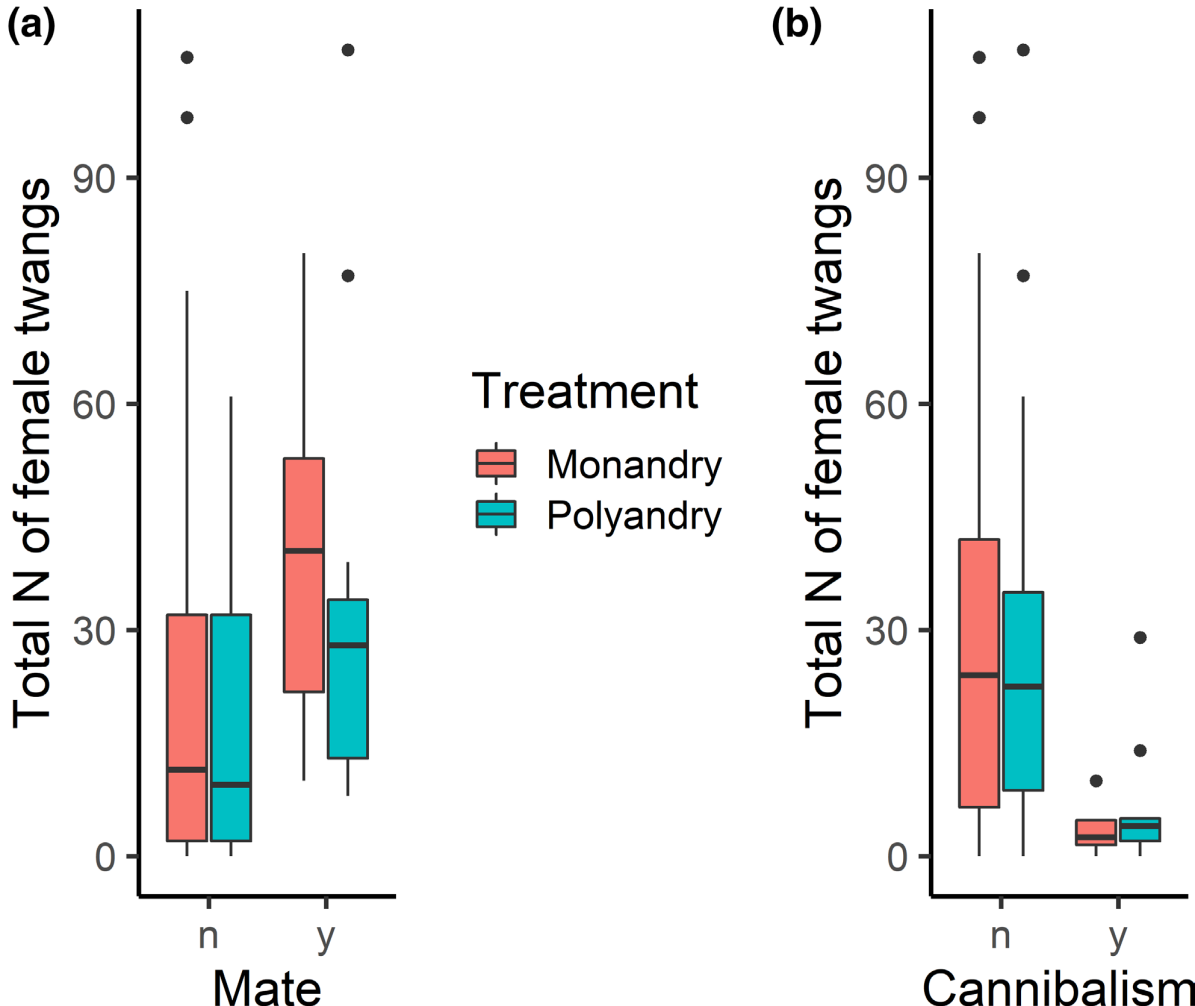
The total number of female twangs (a) during trials with and without successful matings and (b) during trials with and without occurrence of cannibalism in monandrous and polyandrous treatments.

Females performed twanging in 89% of the trials (117 out of 131 video‐scored trials), and males approached females in 76% of the trials (100 out of 131). Males approached females only after females performed twanging (mean time to first female twang, 179.50 ± 25.86 s, *n* = 117; mean time to first male approach 248.58 ± 30.10 s, *n* = 100). The two variables are positively correlated; the sooner the female started twanging the sooner male approached (*R* = .53, *p* < .0001, *n* = 97; Figure 3a) and the total number of female twangs correlate positively with the number of male approaches (*R* = .61, *p* < .0001, *n* = 96, Figure 3b). Higher numbers of female twangs significantly increased mating success (as shown from results of model b in Table 1 and Figure 4a) and reduced the likelihood of cannibalism [GLM—binomial, *β* (95% CI); Intercept −1.29 (−2.19, −0.41); *N* female twangs −0.09 (−0.17, −0.02)]. A short sequence of the mating interaction can be viewed in Video S1.

The number of mating attempts were higher in the trials with successful matings (8.30 ± 1.1, *n* = 43) compared with unsuccessful ones (3.95 ± 0.79, *n* = 84; *t*‐test, *t* = 3.24, df = 81.28, *p* = .0017). In eight trials males entered the mating position twice, meaning they re‐approached the female a second time and were able to couple their pedipalps again during the 20‐min trial, as successful copulation occurred. These were distributed as follows: four trials from the monogamous treatment (three during the trial 1 and one during trial 2) and four trials from the polyandry treatment (two during trial 1 and two during trial 2).

### 3.3 Fitness

One female from the polyandrous treatment died 2 days after the trials, leaving us with a sample of 83 females. The likelihood of successful reproduction (where at least one cocoon hatched) was not affected by the female's mating experience [88.23% mated once (*n* = 30); 66.66% mated twice monandrous (*n* = 4); 87.5% mated twice polyandrous (*n* = 7)], nor by female body mass (Table 5). The total number of cocoons laid by mated females (0–4) within 4 weeks (Table 4) were not significantly affected by the mating experience (once, twice M, twice P), but by female body mass, with lighter females laying more cocoons (Table 5). The proportion of viable cocoons as well as the total number of offspring (Table 4), were not significantly affected by the mating experience, or female body mass (Table 5).

**TABLE 4:** Results from the GLMs investigating the effects of mating experience [mated once and mated twice with the same (monandry) or different males (polyandry)] and female body mass on different measures of fitness.

Model estimates *β* (95% CI)				
	Likelihood of reproduction	Total number of cocoons	Proportion of viable cocoons laid	Total number of offspring
(Intercept)^a^	0.74 (−1.05, 2.47)	3.12 (2.15, 4.03)	0.68 (0.38, 0.98)	226.03 (19.20, 420.07)
Mated twice polyandry	0.98 (−1.89, 3.82)	0.21 (−1.08, 1.52)	0.09 (−0.29, 0.49)	128.66 (−145.81, 405.80)
Mated once	0.98 (−0.99, 3.08)	0.33 (−0.66, 1.36)	0.14 (−0.18, 0.48)	127.51 (−82.01, 347.56)
Female body mass	−3.67 (−37.82, 25.64)	***−13.66 (−26.99, −0.11)***	0.28 (−3.94, 4.11)	−392.33 (−3226.14, 2490.67)

*Note*: Significant effects are shown in bold and in italics.

^a^ Reference category = mated twice monandrous.

**TABLE 5:** Female fitness measures given in mean ± SE, median, range and sample size (*N*).

	Mated once	Mated twice monandry	Mated twice polyandry
Total number of cocoons	3.42 ± 0.23	3 ± 0.63	3.57 ± 0.3
	4, 0–4	3.5, 0–4	4, 2–4
	26	6	7
Proportion of viable cocoons laid	0.82 ± 0.06	0.68 ± 0.17	0.77 ± 0.14
	1, 0–1	0.75, 0–1	1, 0–1
	26	6	7
Total number of offspring	353.38 ± 44.09	224.17 ± 77.95	361.57 ± 114.22
	376, 0–819	263, 0–421	314, 0–921
	26	6	7

*Note*: The columns represent the females that mated once and mated twice with the same (monandry) or different males (polyandry), respectively, throughout the whole experiment.

The number of eggs laid per cocoon and the ratio of hatched eggs (the number of hatched eggs/the total number of eggs per cocoon) per cocoon were not affected by the mating experience (mated once, twice M, twice P), laying order of the cocoon (*n* of cocoon) and/or female mass (Table S4).

## 4 DISCUSSION

Our study was designed to gain insights on the behavior and mating system of the emerging spider model system *Parasteatoda tepidariorum*. We here provide the first qualitative and quantitative description of behaviors that occur during male–female interaction in this species. We show that in our experimental set up matings occurred at very low frequencies, and among those females that mated the majority only mated once, and not necessarily on their first encounter. For the few females that mated twice there was no difference in the likelihood of re‐mating with the same male (monandry) or with a novel male (polyandry). Beyond that, we observed that mating once or twice, regardless of whether monogamous or polyandrous, did not affect female fitness, indicated by similar offspring production in all tested females. In summary, our findings suggest that *P. tepidariorum* possesses a mating system characterized by low mating rates.

### 4.1 Low mating rates in *Parasteatoda tepidariorum*

The low mating rates reported are striking. On the one hand, these may be a consequence of high choosiness of females. Mate choice may provide females with reproductive benefits (Rosenthal & Rosenthal, 2017), despite mate sampling entails energetic costs and the risk of reproductive failure (Kokko & Mappes, 2005). In this respect, one of the general expectations derived from sexual selection theory is, that females are less choosy when unmated, accepting copulations from the first male they encounter to avoid the cost of remaining unfertilized (Andersson & Simmons, 2006; Bleu et al., 2012; Tanner et al., 2019). On the other hand, selection on males to reduce sperm competition may likely be responsible for the evolution of male manipulative adaptations, such as seminal substances that can act on female reproductive behavior and physiology to lower female receptivity (Aisenberg & Costa, 2005; Chapman & Davies, 2004). Interestingly, for *P. tepidariorum* females the likelihood of successful matings did not differ during first or subsequent encounters with males, nor was it affected by the female's mating status (unmated or previously mated). We can therefore exclude unreceptivity and/or enhanced choosiness of once‐mated females as mechanistic explanations for our findings. We, however, cannot entirely rule out that mate choice for traits other than those accounted for in our study may have driven the large numbers of unsuccessful mating attempts. Courtship vibrations in web‐building spiders are known to convey information on the signaler and its underlying quality (Herberstein et al., 2014), lowering female aggressiveness and influencing female mate choice (Wignall & Herberstein, 2013a, 2013b). For example, vibrations performed on cobwebs from widow spiders are suggested to convey information on male size (Sivalinghem & Mason, 2021). We were unable to reliably quantify vibratory movements in the form of male bouncing of the abdomen and/or body rocking, which would have nevertheless not properly reflected how much of the vibratory signals is transmitted to and received by the female, as such mechanical cues depend on several environmental factors.

The lack of successful matings also does not appear to be correlated to lower overall interaction between individuals. Matings occurred in 36% of the trials. However, females signaled in 89% of the trials and males actively approached females in 76% of the trials. Based on our personal observations, together with reports from (Ma et al., 2022), we also note that females occasionally cooperate with males by presenting the opening of their genitalia tract to the courting individual. Yet, we observed that several mating attempts were required prior to successful mating (on average 8) within a 20‐min trial. We cannot exclude that males of this species may require longer interactions with the female, involving repeated mating attempts before successful copulation as these may serve as male courtship and exchange of information between the sexes. Despite an extensive overview of 70 species of Theridiids reported that courtship lasts on average 24 min in comb footed spiders belonging to the Steatoda‐type, which includes *P. tepidariorum*, and 17 min in *P. tepidariorum* (Knoflach, 2004), our experimental setting may not have been optimal and staging longer trials is advisable in future studies.

Ecological constraints that limit the encounter rates between the sexes, such as high mortality and mate‐search costs for males (Byers et al., 2006; Kasumovic et al., 2006), low population densities (Xue et al., 2016), or the spatial and temporal distribution of receptive females (e.g., female‐biased sex ratios; Emlen & Oring, 1977; Ims, 1988; Tuni & Berger‐tal, 2012), may also all contribute to shaping mating systems with low levels of polyandry (Elias et al., 2011). To our knowledge, data on population structure (e.g., sex ratios), genetic variation, densities and natural encounter rates between males and females are missing for this species. We know that mate‐search is risky in spiders (Berger‐Tal & Lubin, 2011; Kasumovic et al., 2006). Male theridids, in particular, develop into adults and leave their webs in search of sedentary females facing high mortality as reported in several widow spiders (Andrade, 2003; Scott et al., 2019; Segev et al., 2003; Segoli et al., 2006). An indication of such costs is for example reported in males of the western black widow *Latrodectus hesperus* that, with an 88% mortality during mate search, parasitize the mate‐search effort of other males in the population to more efficiently find females (Scott et al., 2019). Generally once spider females mature, they signal their receptivity using sex pheromones, emitted from their body or the silk of their webs (Kasumovic & Andrade, 2004; MacLeod & Andrade, 2014). The timeframe of female receptivity is generally short and this imposes further constraints on mating rates, considering also that males generally become sexually mature (i.e., molt to the adult stage) before females (Elias et al., 2011). Low mating rates may also result from monogygy, where males mate with only one female (Schneider & Fromhage, 2010). Such reproductive strategy is facilitated by sexual cannibalism (e.g., self‐sacrifice) and genital mutilation, because of strong sexual selection on competing males (Fromhage et al., 2005), and has been shown to occur in theridids (Michalik et al., 2010). The risk of pre‐copulatory sexual cannibalism, performed in 10% of the trials from well‐fed females in our study, may further act on constraining male mating attempts. Finally, based on our observations and other reports, *P. tepidariorum*, seems to lack forms of male adaptations allowing for female monopolization and limiting inseminations from additional males, such as extremely long copulations that function as mate guarding (Simmons, 2001), or visible plugging of the outer female genitalia (Uhl et al., 2010) which would represent adaptations to high male–male competition. In summary, all the above‐mentioned factors may select for low polyandry in this study species.

### 4.2 Re‐matings do not lead to increased fitness

Low mating rates and monandry may be favored when increased mating rates have little effect on female reproductive output (Klug, 2018). Accounting for the small sample size and in relation to the fitness traits measured in our study, our results suggest that re‐matings did not provide females with either direct or indirect fitness benefits. Indeed, females mated once had similar cocoon and offspring production success as double‐mated females lasting for up to 4 weeks post‐mating. This indicates that females of this species are able to maximize their fitness by mating only once. Despite low sample sizes warrant cautious interpretation, we also found no differences in the reproductive output of females mated monandrously and polyandrously. The benefits of polyandry in spiders have been detected in the form of increased female fecundity (Uhl et al., 2005) or resource acquisition (i.e., foraging advantages; Watson, 1993), but also as benefits to their offspring (Bilde et al., 2007; Watson, 1998; Welke & Schneider, 2009) in several species. We would have expected that any direct benefit derived by mating twice, for example reception of higher amount of sperm and/or ejaculate‐associated components, would be revealed in the form of increased fecundity (i.e., offspring number) for both, monogamous and polyandrous females (Arnqvist & Nilsson, 2000). Yet, only polyandrous females would experience indirect benefits in the form of enhanced fertility (i.e., higher egg hatching), by mating with males of different genetic quality and/or compatibility (Jennions & Petrie, 2000). If the genetic variation in the population is however low, females would fail to derive any indirect benefits from mating polyandrously (Tuni et al., 2012).

Due to lack of visible mating plugs, it appears unlikely—although not impossible—that plugging of the female genitalia during the first mating prevented sperm transfer during the second mating. In contrast to several other spider species which sacrifice their entire pedipalp to plug the females genitalia, *P. tepidariorum* males only can break off the very small and most distal part their pedipalp, which remains inside the female reproductive tract (Abalos & Baez, 1963). Whether this is enough to hinder or lower fertilization success of subsequent matings in females is, however, not known, and would be an interesting venue for future research.

Finally, we found an effect of female body mass on fecundity, as heavier females produced fewer cocoons. This result goes against general predictions of higher body resources being allocated to egg production (Roff, 2002). It may potentially suggest the presence of a trade‐offs, where females invest in egg quality or other traits not measured in our study, at the expense of number of cocoon laid (Reznick, 1992; Stearns, 1992).

### 4.3 Male–female behaviors at mating

The second important outcome of our study is the quantitative description of male and female behaviors at mating. Most ecological studies on *P. tepidariorum* focus primarily on prey capture and web building (Barghusen et al., 1997; Brown & Houghton, 2019; Hajer & Hrubá, 2007; Uma & Weiss, 2012; Valerio, 1975), leaving mating behaviors largely undescribed. Description of mating elements primarily come from two studies. The first—yet based on only three observations—notes that males upload sperm in their pedipalps before copulation through a lengthy process (namely, sperm induction), copulation is obtained by male approach with courtship lasting approx. 17 min, sperm is transferred through a total of two palp insertions, and visible mating plugs do not occur (Knoflach, 2004). In a more recent study, Ma et al. (2022) reported, in undisturbed treatment groups consisting of 20 spider pairs, a 75% mating success, 13.3% of cannibalism, and courtship (defined as the time from male vibratory performance and approach towards the female to the male entering the mating position) lasting on average of 155.5 s. In their study, spiders were left to interact for 1 h but it is not mentioned when males initiated courtship and/or when mating occurred, yet differences in the duration of exposure to the opposite sex may explain the differences with our findings. While cannibalism rates are comparable, mating rates in our study were generally lower and male courtship interactions last for longer. We show that male courtship generally begins with female twanging behavior, where females pluck the web with her two front legs repeatedly, facing the direction of the male. The performance of such web plucking (twang) behavior by females is commonly followed by the male approaching the female and leads to successful mating. Indeed, females performed this behavior more intensively during trials in which males and females eventually successfully copulated. On the contrary, it was weakly performed during trials in which females eventually cannibalized the male. This behavior may be interpreted as a signaling behavior from females that advertise their receptivity. Females that did not have a functional web and were impaired in performing twanging were also not approached by males, indicating that vibrational communication via the web plays a crucial role in male–female reproductive communication in this species (Herberstein et al., 2014), in line with previous observations (Knoflach, 2004; Ma et al., 2022). Males responded to female signaling, by approaching the female, through jerking movements and contact movements of their legs, not always being successful in entering the mating position, as multiple copulation attempts were observed during a single trial (as discussed above). This is an interesting pattern which however differs from knowledge on other comb footed spiders, where it is suggested that males first approach the mate (Knoflach, 2004). Our findings suggest that an individuals' previous social experience affects subsequent behaviors during encounters with a novel individual (i.e., polyandrous treatment). During the second trial females of the polyandrous treatment performed twangs later in time and performed less twangs. This was not affected by their mating status, hence cannot be interpreted as reduced receptivity, but may indicate a more cautious behavior towards novel males. Similarly, and potentially as a consequence, males of the polyandrous treatment approached females later in time and less frequently on their second encounters. There may be higher risks in encounters with novel individuals compared with those previously experienced, as for example the risk of physical injury or harm are unknown (Arnqvist & Rowe, 2013). This interpretation stems from individuals being able to chemically or visually assess and discriminate previous versus novel partners, as described in many arthropods (e.g., female self‐referencing in field crickets; Ivy et al., 2005). Interestingly, this did not translate into a significant delay in mating for polyandrous re‐matings.

Sexual cannibalism occurred primarily before mating, and therefore sets the basis for extreme sexual conflict by preventing successful male reproduction (Schneider, 2014). Pre‐copulatory cannibalism may potentially be driven by female foraging intents, but any fecundity benefit derived from the nutritional value of the male needs to importantly outweigh the costs of remaining unmated for such behavior to be under selection (Welke & Schneider, 2012). It may also be triggered by poor male signaling and/or female mate choice, where preferred phenotypes are allowed to mate, whereas non‐preferred are cannibalized before fertilization (Elgar & Nash, 1988). Interestingly, we show that the relative difference in body mass is higher when cannibalism does not occur, suggesting female foraging preferences for larger males and mating preference for smaller ones. Females spiders may benefit from consumption of males in terms of increase in cocoon mass as shown for *Dolomedes triton* (Johnson, 2005) or fecundity as reported in the mantid *Pseudomantis albofimbriata* (Barry et al., 2008). However, these are examples of species with relatively large males. Fitness benefits of sexual cannibalisms are instead largely lacking in spiders with strong sexual size dimorphism (see list of studies in Barry et al., 2008), where the male soma, even of the larger individuals, may not contribute substantially to the female's diet. In our study system, there may be selection for smaller males, which, despite not being extremely common, has been reported in a various of taxa (Marshal, 1988; Petrie, 1983; Watt et al., 2011), and is often attributed to higher agility and/or lower energy requirements of males possessing smaller bodies (Blanckenhorn, 2000).

## 5 CONCLUSIONS

The low mating rates uncovered in our study may suggest that high selective pressures, potentially reasoned by ecological factors and mating risks for males, are at play to limit mating opportunities. This is further suggested by the lack of fitness benefits derived from multiple matings. While not affecting female reproductive output, matings may carry risks to males due to cannibalistic females. Males may therefore, be under selection to discriminate female receptiveness (Bonduriansky, 2001), most likely through multi‐modal sensory channels, including chemical and vibrational web‐bound communication. Lastly, this study shows that *Parasteatoda tepidariorum* is not only a good developmental model organism amenable to various established techniques to study gene expression and function, but also serves well as a behavioral, neural, and evolutionary model species, given its interesting communication and mating behavior and high numbers of offspring produced by one single mating.

## AUTHOR CONTRIBUTIONS

**Apostolos Angelakakis:** Data curation (lead); formal analysis (lead); investigation (lead); visualization (lead); writing – original draft (supporting). **Natascha Turetzek:** Conceptualization (lead); methodology (equal); project administration (lead); resources (lead); supervision (equal); writing – review and editing (equal). **Cristina Tuni:** Conceptualization (lead); data curation (supporting); funding acquisition (lead); methodology (equal); project administration (lead); resources (lead); supervision (equal); writing – original draft (lead); writing – review and editing (equal).

## FUNDING INFORMATION

CT was funded by the LMUexcellent Junior Research Fund.

## CONFLICT OF INTEREST

The authors have no conflicts of interest to declare.

## Supporting information


Tables S1–S4

Data S1

Video S1

## Data Availability

Data are provided as Supporting Information (Data S1).
