# Supplementary material for: Female mating rates and their fitness consequences in the common house spider Parasteatoda tepidariorum
Source: Ecol Evol. 2022 Dec 28;12(12):e9678. doi: 10.1002/ece3.9678 (PMC9797470; doi:10.1002/ece3.9678)
Supplement: Supplementary file 1 — Tables S1–S4 [file ECE3-12-e9678-s001.docx]

**Appendices**

**Table S1**. Results from the expanded models including male age (N of adulthood days) and male and female spider origin (from wild caught, W and genome line, L) and their interaction, investigating a) the effects of mating treatment (monandry and polyandry), mating trial (1 and 2) and their interaction, and relative body mass between the sexes (female- male mass), on mating success of the pair (GLMM binomial) and **b)** of mating treatment (polyandry and monandry) and the outcome of the previous mating trial (successful, unsuccessful mating) on the likelihood of mating during trial 2 (LM-binomial). Significant effects are shown in bold and in italics.

|  | 1. **Mating success** | 1. **Mating success on trial2** |
| --- | --- | --- |
| **Fixed effects** | **β (95% CI)** | |
| (Intercept)* | -0.74  (-1.75 , 0.36) | -1.23  (-2.46 , -0.02) |
| Treatment (polyandry) | ***1.62***  ***(0.18 , 2.94)*** | 0.07  (-1.07 , 1.24) |
| Mating trial (1,2)^a^ | -0.58  (-1.63 , 0.54) |  |
| Treatment X Mating trial^a^ | -1.23  (-2.78 , 0.34) |  |
| Relative Body Mass^a^ | -2.02  (-19.70 , 14.47) | -0.32  (-19.47 , 19.11) |
| Male origin (W, G) W | 0.003  (-1.39 , 1.33) | 0.09  (-1.39 , 1.63) |
| Female origin (W, G) W | -0.11  (-1.41 , 1.09) | -0.25  (-1.64 , 1.17) |
| Male age^b^ | 0.14  (-0.50 , 0.78) | -0.05  (-0.77 , 0.65) |
| Male Origin X Female Origin | 1.29  (-1.77 , 4.43) | -31.61  (-3299.55 , 3305.04) |
| Previously mated (yes, no) |  | 0.57  (-0.39 , 1.64) |
| **Random Effects** | **σ^2^(Cl 95%)** |  |
| Female ID | 1.21  (0.86 , 1.59) |  |
| Male ID | 0  (0 , 0) |  |

*Reference category, estimate for treatment monandry and mean values of remaining fixed effects

^a^ mean centered

^b^mean-centered and normalized with the standard-deviation

**Table S2.** Results from the GLMMs investigating the effects of mating treatment (monandry and polyandry), trial number (1 and 2), their interaction, and male body mass **(model a)** and intensity of female twanging behavior (N twangs performed) **(model b),** on the likelihood of mating successfully (GLMM binomial). Point estimates and 95% credible intervals are shown on a logit scale and relative to the reference category (Intercept, monandry, and for the other effects in a standardized level). The residual variance component is fixed to π^2^/3. Significant effects are shown in bold and in italics

|  | **Mating success** | |
| --- | --- | --- |
|  | **Model a)** | **Model b)** |
| **Fixed effects** | **β (95% CI)** | |
| (Intercept)* | 0.57  (-1.28 , -0.12) | -0.92  (-1.68 , -0.17) |
| Treatment (polyandry) | 0.75  (-0.21 , 1.72) | 1.09  (-0.03 , 2.19) |
| Mating trial (1,2)^a^ | -0.54  (-1.55 , 0.47) | -0.31  (-1.43 , 0.73) |
| Treatment (polyandry) X Mating trial^a^ | -0.41  (-1.79 , 0.92) | -1.26  (-2.88 , 0.39) |
| Male body size^a^ | -66,16  (-213.49 , 87.59) | -102.91  (-96.92 , 302.29) |
| N female twanging behavior ^b^ |  | ***0.74***  ***(0.29 , 1.16)*** |
| **Random Effects** | **σ^2^(Cl 95%)** | |
| Female ID | 0.47  (0.34 , 0.63) | 0.11  (0.07 , 0.15) |
| Male ID | 0  (0 , 0) | 0.15  (0.11 , 0.19) |

**Table S3.** Results from the expanded models including male age (N of adulthood days) and spider origin (from wild caught, W and genome line, L), investigating the effects of mating treatment (monandry and polyandry), mating trial (1 and 2) and their interaction, relative body mass difference (female - male mass), on male and female mating behaviors. Specifically these are: latency to female twanging (i.e., time from the start of the trial until first female twang); total number of female twangs; latency to first male approach (time from the start of the trial to moving towards the female and making physical contact with front legs); total number of male approaches, as the total number of attempted matings (i.e., male tries to enter the mating position); latency to mating (i.e., time from the start of the trial to successful mating). Significant effects are shown in bold and in italics.

|  | **Latency to female twanging** | **Total N of female twangs** | **Latency to 1^st^ male approach** | **Total N male approaches** | **Latency to mating** |
| --- | --- | --- | --- | --- | --- |
| **Fixed Effects** | **β (95% CI)** | | | | |
| (Intercept)* | 4.85  (4.05 , 5.63 | 3.18  (2.57 , 3.78) | 5.41  (4.69 , 6.17) | 1.63  (0.98 , 2.26) | 415.08  (14.13 , 777.73) |
| Treatment (polyandry) | 0.24  (-0.49 , 0.94) | 0.07  (-0.52 , 0.62) | -0.09  (-0.77 , 0.59) | 0.32  (-0.28 , 0.89) | -12.73  (-306.97 , 282.36) |
| Mating trial (1,2)^a^ | 0.07  (-0.02 , 0.16) | -0.05  (-0.16 , 0.07) | ***0.11***  ***(0.03 , 0.19)*** | ***-0.26***  ***(-0.45 , -0.04)*** | 19.59  (-305.39 , 332.07) |
| Treatment x Mating trial^a^ | ***0.79***  ***(0.67 , 0.92***) | ***-0.47***  ***(-0.70 , -0.24)*** | ***0.89***  ***(0.75 , 1.04)*** | ***-0.61***  ***(-1.02 , -0.19)*** | -286.22  (-853.01 , 267.01) |
| Relative Body Mass^a^ | -9.30  (-22.23 ,  3.57) | 3.20  (-6.49 , 12.62) | 2.56  (-9.14 , 13.81) | 0.11  (-10.25 , 10.13) | -991.48  (-7434.23 , 5585.02) |
| Male origin (W, G) W | -0.87  (-2.11 , 0.39) | -0.41  (-1.46 , 0.63) | -0.71  (-1.92 , 0.51) | -0.12  (-1.24 , 0.97) | 195.39  (-440.84 , 864.15) |
| Female origin (W, G) W | -1.04  (-2.06 , 0.02) | 0.49  (-1.31 , 0.30) | -0.68  (-1.63 , 0.32) | -0.37  (-1.23 , 0.48) | 15.75  (-472.93 , 542.12) |
| Male age^b^ | -0.21  (-0.70 , 0.30) | -0.03  (-0.45 , 0.39) | -0.17  (-0.65 , 0.31) | 0.03  (-0.41 , 0.47) | -286.22  (-174.47 , 353.55) |
| **Random Effects** | **σ^2^(Cl 95%)** | | | | |
| **Female ID** | 0.89  (0.61 , 1.24) | 0.27  (0.18 , 0.37) | 0.48  (0.31 , 0.69) | 0.27  (0.18 , 0.37) | 0  (0 , 0) |
| **Male ID** | 0.95  (0.71 , 1.26) | 0.89  (0.65 , 1.16) | 1.05  (0.75 , 1.43) | 0.80  (0.58 , 1.08) | 17910.29  (8007.63 , 35116.29) |

*Reference category, estimate for treatment monandry and mean values of remaining fixed effects

^a^ mean centered

^b^mean-centered and normalized with the standard-deviation

**Table S4.** Results from the GLMs investigating the effects of mating experience (mated once and mated twice with the same (monandry) or different males (polyandry)) and female body mass on measures of fitness.

|  | **Total number of eggs laid per cocoon** | | **Proportion of viable eggs per cocoon** | |
| --- | --- | --- | --- | --- |
| Fixed effects | **β (95% CI)** | | | |
| **(Intercept)*** | 149.91  (105.45 , 193.59) | 152.18  (107.26 , 197.730 | 0.55  (0.39 , 0.69) | 0.53  (0.38 , 0.68) |
| **Mated twice monandry** | -16.58  (-53.55 , 19.26) | -17.19  (-54.07 , 18.79) | -0.06  (-0.21 , 0.07) | -0.06  (-0.19 , 0.08) |
| **Mated twice polyandry** | 13.17  (-14.88 , 43.02) | 13.82  (-16.03 , 43.67) | -0.10  (-0.21 , 0.02) | -0.11  (-0.22 , 0.008) |
| **Female Body Mass** | 331.38  (-60.62 , 747.57) | 337.78  (-110.97 , 761.99) | 1.37  (-0.17 , 3.00) | 1.32  (-0.40 , 2.95) |
| **N cocoon (1-4)** | -2.19  (-10.79 , 6.34) | -2.26  (-10.29 , 6.02) | 0.02  (-0.004 , 0.04) | 0.02  (-0.002 , 0.04) |
| **Female origin (W, G)** **W** |  | -6.76  (-29.02 , 15.19) |  | 0.06  (-0.03 , 0.14) |
| **Random Effects** |  | **σ^2^(Cl 95%)** | |  |
| **Female ID** | 373.72  (224.89 , 563.97) | 391.48  (236.44 , 599.64) | 0.01  (0.009 , 0.02) | 0.01  (0.008 , 0.02) |

*Reference category, estimate for treatment monandry and mean values of remaining fixed effects
